# Psychometric Properties of the Danish Strength and Difficulties Questionnaire: The SDQ Assessed for More than 70,000 Raters in Four Different Cohorts

**DOI:** 10.1371/journal.pone.0032025

**Published:** 2012-02-27

**Authors:** Janni Niclasen, Thomas William Teasdale, Anne-Marie Nybo Andersen, Anne Mette Skovgaard, Hanne Elberling, Carsten Obel

**Affiliations:** 1 Department of Psychology, University of Copenhagen, Copenhagen, Denmark; 2 Department of Public Health, University of Copenhagen, Copenhagen, Denmark; 3 Child and Adolescent Psychiatric Centre Glostrup, Copenhagen University Hospital, Copenhagen, Denmark; 4 Department of Public Health, Aarhus University, Aarhus, Denmark; The University of Queensland, Australia

## Abstract

**Background:**

The Strength and Difficulties Questionnaire (SDQ) is a brief behavioural five factor instrument developed to assess emotional and behavioural problems in children and adolescents. The aim of the current study was to evaluate the psychometric properties for parent and teacher ratings in the Danish version of SDQ for different age groups of boys and girls.

**Methods:**

The Danish versions of the SDQ were distributed to a total of 71,840 parent and teacher raters of 5-, 7- and 10- to 12-year-old children included in four large scale Danish cohorts. The internal reliability was assessed and exploratory factor analyses were carried out to replicate the originally proposed five factor structure. Mean scores and percentiles were examined in order to differentiate between low, medium and high levels of emotional and behavioural difficulties.

**Results:**

The original five factor structure could be substantially confirmed. The Conduct items however did not solely load on the proposed Conduct scale and the Conduct scale was further contaminated by non-conduct items. Positively worded items tended to load on the Prosocial scale. This was more so the case for teachers than for parents. Parent and teacher means and percentiles were found to be lower compared to British figures but similar to or only slightly lower than those found in the other Nordic countries. The percentiles for girls were generally lower than for boys, markedly so for the teacher hyperactivity ratings.

**Conclusions:**

The study supports the usefulness of the SDQ as a screening tool for boys and girls across age groups and raters in the general Danish population.

## Introduction

Mental health problems of children and adolescents occur frequently in the general population with prevalence rates of psychopathology estimated from 7% in rural Brazil and Norway, 10% in Britain and Denmark and up to 15% in Russia and Bangladesh [1]–[6]. In Denmark one prevalence study indicated that approximately 10% of Danish 8–9-year-olds meet the DSM-IV criteria for a psychiatric diagnosis [7]. However, a considerable discrepancy has been found between prevalence rates and the number of children being treated through childhood and adolescence. This is disturbing as psychopathology developed in childhood shows stability over time and can progress into adult psychiatric disorders. Factors associated with the development of psychopathological disorders include age and gender, socioeconomic markers and family conditions [8]. The strength of these associations may however vary between cultural settings. In order to screen for mental health disorders in children and adolescents there is a need for instruments to assess for behavioural and emotional problems, which have been validated across cultural settings.

The strengths and difficulties questionnaire (SDQ) is a brief instrument developed to screen for child and adolescent psychopathology. It is used worldwide, has been translated into more than 60 languages, and has screening properties comparable with more comprehensive instruments [9]. It consists of 25 items and generates scores within five domains of psychological adjustment: Hyperactivity/Inattention (hereafter Hyperactivity), Peer problems, Conduct problems, Emotional symptoms and Prosocial behaviours. The items are based on key symptoms for DSM-IV diagnoses and have been grouped into scales using exploratory factor analysis. The five hyperactivity items have for example been selected to assess hyperactivity, inattention and impulsiveness as these constitute the key symptoms for the DSM diagnosis of Attention/Deficit Hyperactivity Disorder (ADHD). The questionnaire is widely used for clinical as well as research purposes [10].

The SDQ appeals to researchers as well as clinicians for several reasons: firstly, because of its brevity, secondly because it covers key aspects of common childhood and adolescence psychopathology, and thirdly because it includes strengths as well as difficulties, which makes it more acceptable for parents, especially in the general population.

The SDQ has been used extensively in European as well as non-European contexts [10], [11] since it was developed by Goodman in Britain in the late 1990s as an extension of the early work of Rutter [12]. A recently published review looking into the psychometric properties of the parent and teacher versions of the SDQ included 48 studies from 17 different cultural settings and a total of 131,223 raters [11]. Mean scores and cut-offs have shown some variation across cultural settings indicating some variations in the prevalence of child and adolescence psychopathology. British presented mean scores and cut-offs tend to be higher than northern European mean scores [13], but similar to or lower than the mean scores presented for the southern European countries [14]. Outside European settings, markedly higher than British mean scores have been reported for (non-western) Chinese and Brazilian children [3], [15] but similar to American and Australian samples [16], [17].

Most studies looking into the factor structure of the SDQ have applied exploratory factor analyses (EFA) and Principal component analysis (PCA). These have by and large found support for Goodman's predicted five factor model [15], [18]. Few studies have applied confirmatory factor analysis (CFA) and those that have done so have not found unequivocal support for the five factor model [19]–[21]. Dickey and Blumberg found support for a three factor structure representing prosocial, internalising and externalising problems in an American sample of 4–17-year-olds and concluded that U.S. parents may construe conduct and peer problems differently from European parents [19]. Along the same lines, a British study concluded that there are advantages to using the broader internalising and externalising subscales for analyses in low-risk epidemiological samples, while one should retain the five subscales when screening for disorders among high-risk children [20]. On the other hand one thorough Norwegian study applying both confirmatory and exploratory factor analyses found none of the alternative models to fit the data better than a slightly modified version of Goodman's five factor model [21].

The discrepancies found in the existing literature for the mean scores and cut-offs, as well as for the factor structure therefore need further investigation. Culture plays a major role in the expression of psychosocial problems and for this reason previous investigations of discrepancies between studies have not been able to identify the extent to which they are expressions of true differences in scores and to what extent they are caused by demographic or cultural variations. In order to rule out any potential cultural and linguistic factors there is therefore a need for a study looking further into these variables within a homogenous cultural and linguistic setting from a large number of raters. Such study would also permit for cross-age, cross-gender and cross-rater comparisons.

Several large scale birth cohorts have been established in Denmark within the last few decades, a number of which have included the SDQ in their follow-up phases. Denmark may therefore, despite its small size, be the country in which the largest number of SDQ ratings has been collected. The aims of the current study were therefore: 1. to evaluate the internal reliability and the five domains of psychological adjustment supposedly evaluated by the SDQ by means of principal component analysis, and 2. to evaluate the mean scores and percentiles across age groups, gender and raters. This is performed for parent and teacher raters, boys and girls and 5-, 7- and 10–12-year-olds separately. It is hypothesised that sound reliability will be established, particularly so for the hyperactivity scale, and that the original proposed five scale factor structure will be confirmed. It is further hypothesised that mean scores and percentiles will be similar to those found in the other Nordic countries but lower compared to other European settings.

## Methods

### Samples

Included in the present study are data from four general population-based, large-scale birth cohorts, namely the Copenhagen Child Cohort (CCC2000), the Danish National Birth Cohort (DNBC), the Danish National Institute of Social Research's (DNISR) and the Aarhus Birth Cohort (ABC). Data come specifically from the 5-year follow up of the CCC2000, the 7-year follow up of the DNISR and DNBC and the 10–12-year-old of the ABC. Specific participation characteristics of the individual cohorts are shown in Table 1. Details of the methodology of the individual cohorts have been described in more detail elsewhere [22]–[25]. Drop-out rates were found to vary between cohorts. However, despite different drop-out rates all cohorts had contact information on most participating women (>99%). Thus, most of the non-participating women were non-responders. Compared to the background population the samples were under-represented regarding low socioeconomic resources (education, occupation, income and civil status), parents who were not born in Denmark; younger mothers; low maternal education; parents living separately at the time of birth; and changed family composition in the first five years of life [23], [26]–[28].

**Table 1:** Characteristics of the birth cohorts providing SDQ data for the study.

Cohort	Copenhagen Child Cohort	Danish National Birth Cohort	Danish National Institute of Social Research	Aarhus Birth Cohort
Acronym	CCC2000	DNBC	DNISR	ABC
Recruitment period	2000	1996–2002	1995	1990–1992
Study population: Eligible for the included follow-up	5,898	83,315*	5,233	8,244
Parent contribution of SDQ	3,349 (57%)	48,544 (58%)	4,971 (95%)	6,751 (82%)
Teacher contribution of SDQ	2,594 (44%)	-	-	5,631 (68%)
Age at SDQ screening	5	7	7	10–12

*As per October 2009.

The department where the study was carried out did not have an internal review board. However, the collection and analysis of data from the four databases was in each case approved of by regional ethic committees - De Videnskabsetiske Komiteer for Region Hovedstaden for CCC2000, DNBC and DNISR and De Videnskabsetiske Komiteer for Region Midtjylland for ABC. The parents and teachers in each of the four cohorts were in writing made aware that the data was used for research purposes and verbally gave their consent for the data being used for these purposes. The parent consent was required before any approach was made to the child's teacher. The regional ethics committees approved the use of these verbal informed consent procedures for each cohort.

### Materials

The SDQ contains 25 questions and an Impact supplement. The 25 questions ask about different positive and negative aspects of the child's behaviour, and can be scored *‘not true’*, *‘somewhat true’* and *‘certainly true’*. Of the 25 questions, 10 are generally thought of as strengths, 14 as difficulties and 1 as a neutral question. The items are divided into five scales (*Hyperactivity, Emotional, Conduct, Peer problem and Prosocial*) of five items each [12]. The first four scales are summed to obtain a total difficulties score whereas the Prosocial scale was included in order to enhance acceptability on part of the rater [12]. The questions have been selected on the basis of contemporary nosological concepts as well as factor analytically derived dimensions [12], [18]. An extra Impact supplement begins with one screening question asking whether the rater *“overall thinks that the child has difficulties in one or more of the following areas: emotions, concentration, behaviour or being able to get on with other people”*. If the rater answers *“yes”* to this question further items inquire about the severity of these difficulties. The Impact supplement provides an important estimate of the burden of the problems which is an essential part of the diagnostic criteria in the current diagnostic classification systems, ICD-10 and DSM-IV [12], [18]. The Danish parent and teacher versions were translated in 2001, implementing standard back-translation procedures and using concepts and terms that were in keeping with time [29].

### Statistical analyses

Analyses were carried out using the statistical package SPSS 18 and were conducted on unweighted data. Employed methods include scale reliability analyses, exploratory factor analysis by means of Principal Component Analysis and descriptive statistics. Because of the non-normal distribution of data all statistical group comparisons were carried out by means of the Mann-Whitney's U-test. For the sake of uniformity, responses of five items which were otherwise scored in a positive direction were inverted prior to their inclusion in the different analyses, and the item order was rearranged for visualisation purposes.

## Results

### Missing data

Goodman suggests that cases be included only when a minimum of three answers are given on any single scale [18]. In the present study the problem of missing values proved to be small and it was for this reason decided to include cases with a total of not more than one missing value. The employed sample sizes were thus 3,349 and 2,594 for parents and teachers of 5-year-olds, 53,515 for parents of 7-year-olds and 6,751 and 5,631 for parents and teachers of 10–12-year-olds comprising a total of 71,840 raters. In all parent samples there was a small overrepresentation of boys whereas there was a small overrepresentation of girls in the two teacher samples (app. 51/49%).

### Validation of the scales

Initially, response frequencies for each of the 25 individual items were examined. It appeared that all items for all samples and raters were non-normally distributed with highly positively skewed distributions, especially so for the Conduct and Peer problem items. Particularly skewed were the two conduct items *“fights”* and *“steals”* with only 0.6 and 0.3% of responders agreeing the item to be *“certainly true”* and between 95.6% and 98.1% declaring it *“not true”*.

In order to determine the construct validity of the SDQ inter-item correlations were computed for the 7-year sample. All 20 problem-items as well as the five prosocial items were found to be positively correlated with each other which preliminary indicates that a single latent variable may influence the individual item responses. To further test this hypothesis Cronbach's Alphas were calculated including the option “scale if item is deleted”. A higher Alpha appeared from these analyses only for the item *“somatic”* on the Emotional scale indicating that this item may cause some problem for the validity of the scale. However as it was only marginally higher (0.615 and 0.627) it was decided to retain the item for the remaining analyses.

### Reliability

Cronbach's Alphas were also calculated for each subscale, the Total difficulties and the Impact score, individually for each subgroup, for parent and teacher raters separately and for boys and girls separately. Notwithstanding the fact that SDQ subscales only comprise five items, the coefficients were generally considered high. Highest estimates were found for the Hyperactivity scale (0.73–0.86) and for the 20 item Total difficulties scale (0.75–0.88) and lowest estimates for the Conduct scale (0.44–0.73). Reliabilities were generally found to be higher for boys than for girls and typically higher for teacher ratings compared to parent ratings for the individual subscales and total difficulties score, but lower so for the impact scores. These somewhat lower reliabilities for the Impact score may be broadly a result of the fact that teacher estimates are calculated on the basis of only three items whereas parent estimates are based on five items.

### Inter-rater reliability

The 5- and 10–12-year-old dataset further allowed for an exploration of inter-informant correlations between parents and teacher ratings. For 5-year-olds Pearson's Product moment correlations were found to be: Hyperactivity: 0.42; Emotional: 0.33; Conduct: 0.33; Peer: 0.37; Prosocial: 0.29; Total difficulties: 0.45 and Impact: 0.41. For the 10–12-year-olds the corresponding figures were: Hyperactivity: 0.50; Emotional: 0.37; Conduct: 0.37; Peer: 0.49; Prosocial: 0.30, Total difficulties: 0.53 and Impact: 0.50. Comparing younger and older children it appears from the higher correlations for 10–12-year-olds that parent and teachers consistently rate older children more similar than younger ones.

### Factor Structure

Since the internal consistency of the individual subscales and total difficulties scale were considered high, the next step was to determine the dimensionality of the SDQ. Principal component analyses (PCA) with Promax Rotation was carried out. Promax rotation was chosen as this rotation technique is particularly useful for large datasets. It was also chosen as it allows for correlations between factors and it produces both a pattern matrix and a structure matrix both of which are presented below. The values of the structure matrices are presented as they illustrate correlations between items and factors. The values of the pattern matrices are however also presented as they are similar to the easily interpretable values obtained in orthogonal rotations presented by most other researchers.

The analyses were firstly run separately for boys and girls for each of the four samples. The initial PCA analyses showed that the items generally loaded on the same factors between age-groups and gender. For this reason it was decided to pool the data into a large parent sample and a large teacher sample and run the analyses separately for these two groups. The extraction of the PCA were initially based on the number of Eigenvalues greater than 1 which resulted in a five factor solution for parents but a six factor solution for teachers. However, since the sixth factor had an Eigenvalue of 1.008 and only accounted for 4.03% of the variance it was decided to omit this factor from any further analyses and to run the analyses specifying the number of factors to be five.

It appears from Tables 2 and 3 that virtually all 25 items showed the highest loadings on their respective proposed scales. Teacher ratings showed higher loadings on their respective scales than did parent ratings. The values of the pattern matrices for both parents and teachers showed unequivocal high loadings on their proposed scales. The structure matrices on the other hand showed a somewhat more ambiguous picture. Conduct items showed high loadings on the other scales and non-conduct items loaded on the Conduct scale. Positively worded items further loaded on the Prosocial scale. This was more so for teacher raters compared to their parental counterparts.

**Table 2:** Principal Component Analysis with Promax rotation for parent ratings.

Parents 5–12-year-olds (N = 61,789)					
Principal component	1	2	3	4	5
Initial Eigenvalue:	4.51	2.00	1.65	1.33	1.19
Initial explained variance	18.04	8.00	6.58	5.31	4.76

Extracted Factors	Hyperactivity	Prosocial	Emotional	Peer	Conduct
Restless	**.75 (.77)**				
Fidgety	**.72 (.77)**				
Distracted	**.77 (.80)**				
Reflects	**.59 (.51)**	−.41			
Attends	**.74 (.78)**				
Somatic			**.41 (.42)**		
Worries			**.68 (.68)**		
Unhappy			**.61 (.55)**		
Clingy			**.68 (.69)**		
Afraid			**.70 (.71)**		
Tantrum					**.54 (.47)**
Obedient	.43	−.41			**.45 (.31)**
Fights				.41	**.55 (.47)**
Lies					**.66 (.67)**
Steals					**.53 (.64)**
Loner				**.62 (.62)**	
Friend				**.50 (.56)**	
Popular				**.62 (.58)**	
Bullied				**.53 (.44)**	.40
Oldbest				**.68 (.68)**	
Considerate		**.63 (.54)**			−.43
Shares		**.57 (.56)**			
Caring		**.67 (.70)**			
Kind		**.50 (.53)**			
Helpout		**.64 (.69)**			

Data from the Structure matrix is presented with data from the Pattern matrix in brackets. Factor loadings between +/−0.4 omitted. The bolded items show the proposed factor loadings.

**Table 3:** Principal Component Analysis with Promax rotation for teacher ratings.

Teacher 5–12-year-olds (N = 6,829)					
Principal component	1	2	3	4	5
Initial Eigenvalue:	7.22	2.80	1.80	1.36	1.15
Initial explained variance	28.88	11.22	7.19	5.42	4.59

Extracted Factors	Hyperactivity	Prosocial	Peer	Emotional	Conduct
Restless	**.84 (.89)**				.45
Fidgety	**.80 (.88)**				
Distracted	**.84 (.91)**				
Reflects	**.73 (.56)**	−.57			.49
Attends	**.80 (.81)**	−.48			
Somatic				**.55 (.55)**	
Worries				**.75 (.75)**	
Unhappy			.46	**.70 (.63)**	
Clingy				**.71 (.67)**	
Afraid				**.74 (.76)**	
Tantrum	.42		.44		**.67 (.57)**
Obedient	.64 (.43)	−.51			**.55 (.26)**
Fights	.48	−.46			**.77 (.67)**
Lies	.41				**.73 (.74)**
Steals					**.55 (.70)**
Loner			**.72 (.75)**	.41	
Friend			**.76 (.82)**		
Popular		−.54	**.72 (.59)**		.49
Bullied			**.62 (.59)**		
Oldbest			**.75 (.81)**		
Considerate	−.51	**.79 (.65)**			−.54
Shares		**.75 (.72)**			−.40
Caring		**.81 (.86)**			
Kind		**.73 (.77)**			
Helpout		**.76 (.88)**			

Data from the Structure matrix is presented with data from the Pattern matrix in brackets. Factor loadings between +/−0.4 omitted. The bolded items show the proposed factor loadings.

### Mean scores and percentiles

Since the internal consistencies were found to be high and the factor structure could substantially be confirmed for boys and girls, younger and older children and parent and teacher ratings it was decided to examine any potential differences in scores between these groups. Tables 4 and 5 present the means and standard deviations (SD) for each of the five subscales, the Total difficulties and Impact scores for parent and teacher raters respectively. For each sample it appears that girls scored higher than boys on the Emotional and Prosocial subscales whereas boys scored higher on the Externalising (Conduct and Hyperactivity) and Peer scales. Parent and teachers alike rated older children as exhibiting fewer hyperactive and conduct problems and with more prosocial skills compared to younger ones. Teachers furthermore rated older children as also having more peer problems compared to younger ones. The statistical significance of these differences was examined using Mann Whitney-U tests. As could be expected given the very large sample sizes, most comparisons proved to be statistically significant (P<0.05). The effect sizes (Cohen's D) were found to be of medium size for the Hyperactivity, Prosocial and Total difficulties for all age groups and raters and also of medium size for teachers. Teachers generally rated girls and boys more dissimilarly than parents.

**Table 4:** Mean sum scores and Standard deviations for 5-, 7- and 10–12-year-old parent ratings.

	5-year-olds (N = 3,288)						7-year-olds (N = 53,476)						10–12-year-olds (N = 5,031)					
Parents	Boys		Girls		Gender effects		Boys		Girls		Gender effects		Boys		Girls		Gender effects	
SDQ scale	Mean	SD	Mean	SD	P-values	Cohen's D	mean	SD	mean	SD	P-values	Cohen's D	mean	SD	mean	SD	P-values	Cohen's D
Hyperactivity	2.74	2.27	2.11	1.94	0.000	0.30	2.73	2.31	2.02	1.98	0.000	0.33	2.35	2.25	1.71	1.88	0.000	0.31
Emotional	1.58	1.73	1.59	1.75	0.930	0.01	1.59	1.75	1.66	1.76	0.000	0.04	1.59	1.90	1.72	1.88	0.000	0.07
Conduct	1.32	1.43	1.12	1.27	0.000	0.15	1.28	1.35	1.15	1.21	0.000	0.10	0.98	1.29	0.84	1.12	0.001	0.12
Peer	0.97	1.57	0.79	1.33	0.002	0.12	0.82	1.36	0.63	1.13	0.000	0.15	0.99	1.59	0.89	1.52	0.001	0.06
Prosocial	8.02	1.66	8.57	1.45	0.000	0.35	8.09	1.66	8.69	1.40	0.000	0.39	8.21	1.70	8.80	1.38	0.000	0.38
Total Difficulties	6.60	4.97	5.61	4.26	0.000	0.21	6.41	4.77	5.44	4.16	0.000	0.22	6.85	6.48	4.54	5.25	0.000	0.39
Impact	0.29	1.06	0.14	0.69	0.000	0.17	0.26	0.95	0.13	0.65	0.000	0.16	0.49	1.07	0.27	0.79	0.000	0.24

Gender effects show the 2-tailed p-values (Mann-Whitney U tests) with effect sizes (Cohen's D).

**Table 5:** Mean sum scores and Standard deviations for 5- and 10–12-year-old teacher ratings.

	5-year-olds (N = 2,542)						10–12-year-olds (N = 4,264)					
Teachers	Boys		Girls		Gender effects		Boys		Girls		Gender effects	
SDQ scale	Mean	SD	Mean	SD	P-values	Cohen's D	mean	SD	mean	SD	P-values	Cohen's D
Hyperactivity	2.96	2.85	1.68	2.31	0.000	0.50	3.03	2.96	1.39	1.99	0.000	0.66
Emotional	1.31	1.76	1.41	1.79	0.112	0.06	1.28	1.93	1.43	2.00	0.003	0.08
Conduct	1.24	1.74	0.75	1.35	0.000	0.32	1.13	1.73	0.51	1.15	0.000	0.43
Peer	0.99	1.71	0.82	1.46	0.019	0.11	1.41	1.99	1.21	1.92	0.000	0.10
Prosocial	7.19	2.37	8.34	1.90	0.000	0.54	6.75	2.62	8.10	2.09	0.000	0.57
Total Difficulties	6.50	5.87	4.67	4.98	0.000	0.34	6.85	6.48	4.54	5.25	0.000	0.39
Impact	0.33	0.83	0.19	0.66	0.000	0.19	0.49	1.07	0.27	0.79	0.000	0.24

Gender effects show the 2-tailed p-values (Mann-Whitney U tests), with effect sizes (Cohen's D).

Following Goodman's recommendations with approximately 80% of children defined as being within a “*normal*” range, 10% in a “*borderline*” range and the highest 10% grouped in an abnormal or “*clinical*” range these percentiles were then calculated for the samples of 5–7- and 10–12-year-olds [12]. The upper percentile for the Total difficulties scores were for boys and girls in the present study found to be between 11 and 14 for parent ratings and between 12 and 18 for teacher ratings. As anticipated on the basis of the mean scores presented above, girls were generally rated as having fewer difficulties than boys, contributing to a broader range of scores for girls in the clinical percentile. This difference was particularly noticeable on the Hyperactivity scale which also contributed to the differences in Total difficulties score. Girls on the other hand had a narrower band of scores in the Prosocial banding indicating higher prosocial ratings. Comparing teacher with parent ratings the differences in scores on the Hyperactivity scale were even more marked, indicating that teachers are more likely to rate boys differently than girls differently on this scale (please contact the first author for a table with the full details of the distribution of ranges and percentiles).

The percentiles were also compared to Goodman's British scores. For the Total difficulties scores the British *“clinical”* percentiles were found to be 17 for 5–15-year-old boys for parent as well as teacher raters but 15 and 12 for girls for parent and teacher raters respectively. Applying the parent scores of 17 and 15 for boys and girls respectively only included between 2.9% and 4% of the children in the present cohorts. The scores for teacher of 17 and 12 for boys and girls on the other hand included a larger proportion of the children, namely between 8.3% and 6.3% of the samples thus being more similar to the Danish distribution of scores.

## Discussion

This article presents the psychometric properties of the Danish SDQ from a total of 71,265 raters after excluding data on the basis of missing values. To the authors' knowledge this is the first time that data from so many informants from the same cultural setting have been included in the same study. By contrast, a recently published review presented results from 48 studies from across the world with a total of 131,223 raters [11]. This review noted that the methodologies of the included studies varied making it difficult to compare them. Strengths of the present study are the inclusion of studies that apply similar methodologies and are derived from the same cultural setting creating a unique opportunity to investigate the psychometric properties of the SDQ between genders, ages and raters. It appears from the above presented analyses that the psychometric properties of the Danish version of the SDQ are strong, particularly for the teacher version.

The pattern matrices of the EFA replicated Goodman's five factor structure for parents and teachers. It appears from the higher teacher loadings that the questionnaire works a little better for teachers than for parents. Investigating the structure matrices, however, revealed two kinds of scale problems that are worth mentioning: firstly, that Conduct items load on non-conduct scales and conversely non-conduct items load on the Conduct scale and secondly that the positively worded items tend to load on the Prosocial scale. This is more so for teachers than for parents. With regard to the high loading of the Conduct items on the other scales it seems that these items are as much part of a hyperactivity construct as part of a notion of conduct for teachers. This is somewhat in line with a British study [20] applying CFA that concludes that the five subscales may not tap into distinct aspects of child mental health among low-risk, epidemiological samples which is exactly what characterises the four included samples. Instead one should use the broader Internalising and Externalising subscales. In regards to the positively worded items this finding is in line with Goodman [18] who also found these items to load on the Prosocial scale. Although the positively worded items are precisely one of the advantages of this questionnaire they also seem to involve some psychometric drawbacks. Thus, although the five dimensions could overall be confirmed by examination of the pattern matrix (indicating no scale problems) the distinctiveness of the factors and some of the items do not seem particularly strong when one investigates the structure matrix that allows for cross-loadings between factors. For the clinicians this means that one should not put too much emphasis on the five individual subscales, much less use the SDQ as a diagnostic tool. These rater differences also illustrates the importance of running rater specific analyses.

The reliability estimates presented above are very similar to those found in other studies [11]. Sound reliability estimates and factor loadings of the hyperactivity scale indicate that the SDQ provides a solid estimate of symptoms of ADHD. The reliability of the Emotional scale has generally been reported as being poorer than what was found in this study, indicating that Danish parent and teachers may be better at reporting Internalising problems compared to other cultural settings. The Conduct subscale was, on the other hand, uniformly found to have the lowest reliability estimates and the lowest factor loadings, indicating a limitation of the usefulness of the scale within a low risk sample.

Lower reliability estimates were found for parents compared to teachers indicating that teachers are more likely than parents to view individual subscale items as measuring the same ability or trait. This may indicate that the subscale items may be viewed as less one-dimensional by parents caused by different tester attitudes. Conversely, teachers may be influenced by some sort of *“halo-effect”* which in the literature is referred to as the impact of one class of behaviour on the perception of another [30]. This means that children exhibiting problem behaviours in one area are more likely to be rated as problematic in other areas as well. Support for this hypothesis also comes from the teacher factor loadings where several items show high loadings on more than one subscale. Halo-effects have in the literature been found to show a different pattern for boys and girls and these tendencies could also contribute to the higher reliability estimates for boys than for girls [30].

The means and percentiles presented above are in line with those reported for other Scandinavian studies and somewhat lower on the Hyperactivity, Peer and Total difficulties scales compared to those found in other European and non-European studies [10], [11]. The 90^th^ percentile for the Total difficulties scores were for boys and girls in the present study found to be between 11 and 14 for parent ratings and between 12 and 18 for teacher ratings. These parent ratings are somewhat lower than the British recommendation of 17 [18] and Swedish of 14 [31] indicating that children of all the included age groups are rated as exhibiting fewer emotional and behavioural problems compared to other samples. Different explanations for the above described differences can be given. Firstly, they may indicate that Danish parents and teachers rate children more positively than do British parents and teachers. When the upper 10% British percentiles for boys and girls were applied for parent and teacher raters it appeared that the teacher ratings were more similar across cultures than the parent ratings indicating that this is only so for the parents. Secondly, it may be that the included samples are more selective and therefore less representative of the general population compared to the samples included in other studies. The present study is characterised by four large scale cohorts with attrition rates between 5 and 56% making the samples more or less non-representative of the general population biasing the included children toward a psychiatrically low-risk sample. This was particularly true for the large DNBC cohort. Since data were included in the analyses without compensatory weightings for underrepresented groups this may have introduced a potential source of bias. Thirdly, it may reflect actual behavioural and emotional differences in the Nordic countries characterised by better social security, low poverty, high living standards and less economic and social inequality. Meltzer et al. [8] found that children with mental disorder were more likely to live in lower income households, with a lone parent and in social sector housing. Denmark is characterised by a relatively homogenous population with a high level of social security which may cause fewer behavioural and emotional problems in the general populations.

Looking into potential gender differences boys were found to score higher than girls on the Hyperactivity, Conduct and Peer subscales and Total difficulties and Impact scores. Girls on the other hand were rated higher on the Emotional and Prosocial scales. Few other studies have reported potential significant differences between boys and girls [32]. The present study found medium to large effect sizes between boys and girls on the Hyperactivity, Conduct and Prosocial scales and Total difficulties scores. The present large-scale study has thus shown the importance of running the analyses separately for boys and girls. Failure to do so may potentially mask large differences between the sexes.

Younger children were in the present study found to score higher than older ones on the two Externalising subscales (Hyperactivity and Conduct scales). This is in similar vein to a German study [33] reporting younger children exhibiting more hyperactivity compared to older ones and a Dutch study [34] reporting a decline in parent ratings of total difficulties, emotional and hyperactivity scores with age. Interestingly, this same study reported increased total difficulties, conduct and emotional scores for teacher ratings as compared to parental ratings. Again, these results show the importance of running separate analyses for younger and older children.

Some limitations of the present study should be noted. The questionnaires from all the cohorts were mainly completed by mothers rather than fathers and this may have had an impact on the distribution of scores. Generally, other studies do not report on the gender distribution of the rater and this may cause some of the variability of scores across studies. Additionally, future studies would benefit from including information on socioeconomic risk factors. One study did find strong effects of social class on the Hyperactivity scale and somewhat less on the Peer scale [33] so controlling for a number of risk factors as for example second order factors in confirmatory factor analyses will further improve the findings of future studies. Future studies should further investigate different factor models using a confirmatory factor analytic approach. Finally, the SDQ is a widely used instrument in Danish epidemiologic studies and future work could advantageously examine the predictive validity of the SDQ as this is of prime importance in order to know how well the SDQ predicts future child, adolescent and adult psychiatric illness.

In conclusion, despite the above mentioned limitations this study demonstrates that SDQ is a well-functioning questionnaire with sound psychometric properties. The internal consistency is high, the factor structure could largely be confirmed and the means and percentiles make theoretical sense.
